# Spontaneous and Treatment-Related Changes of Serum Calcitonin in Medullary Thyroid Cancer: Long-Term Experience in a Patient With Multiple Endocrine Neoplasia Type 2B

**DOI:** 10.1200/PO.23.00675

**Published:** 2024-05-06

**Authors:** Zsuzsanna Réti, Ádám Gy. Tabák, Miklós Garami, Ildikó Kalina, Gergely Kiss, Zoltán Sápi, Miklós Tóth, Judit Tőke

**Affiliations:** ^1^ Department of Internal Medicine and Oncology, Semmelweis University, Faculty of Medicine, Budapest, Hungary; ^2^ George Emil Palade University of Medicine, Pharmacy, Science, and Technology of Targu Mures, Târgu Mureş, Romania; ^3^ Department of Public Health, Semmelweis University, Faculty of Medicine, Budapest, Hungary; ^4^ UCL Brain Sciences, University College London, London, United Kingdom; ^5^ Pediatric Center, Semmelweis University, Faculty of Medicine, Budapest, Hungary; ^6^ Medical Imaging Centre, Semmelweis University, Faculty of Medicine, Budapest, Hungary; ^7^ Department of Pathology and Experimental Cancer Research, Semmelweis University, Budapest, Hungary

## Abstract

**PURPOSE:**

Medullary thyroid carcinoma (MTC) in MEN2B syndrome is associated with germline *RET* mutation. Patients harboring de novo mutations are usually diagnosed at more advanced disease stages. We present a young woman with Met918Th mutation diagnosed with stage IV MTC at age 10 years.

**METHODS:**

The disease progressed despite total thyroidectomy and multiple surgical interventions for cervical lymph node recurrences, leading to distant metastases in the fifth year after the initial diagnosis. Subsequently, she underwent five different types of tyrosine kinase inhibitor (TKI) treatments. The 17-year disease course was divided into periods defined by four surgical interventions and sequential treatment intervals with four multikinase (sunitinib, vandetanib, cabozantinib, and lenvatinib) and one RET-selective TKI (selpercatinib). Tumor growth for different phases of spontaneous development and drug treatment intervals was characterized by changes in serial log-transformed calcitonin measurements (n = 114).

**RESULTS:**

Three operations (one for calcitonin-producing adrenal pheochromocytoma) were associated with drops in calcitonin levels. All of the nonselective TKIs were stopped due to adverse effects. As reflected by the negative calcitonin doubling rate, the best treatment response was observed with selpercatinib, which was associated with an initial large drop followed by a decreasing calcitonin trajectory over 514 days without any major side effects.

**CONCLUSION:**

This case of MEN2B medullary thyroid cancer with long-term survival presents how the effectiveness of different treatment modalities can be estimated using log-transformed calcitonin levels. Furthermore, our experience supports the view that serial calcitonin measurements may be more sensitive than radiological follow-up in advanced MTC. Our patient also represents a new case of rarely reported calcitonin-producing pheochromocytomas.

## Teaser

Case report by Zs. Reti et al from @semmelweishu on MEN2B-related advanced MTC highlights the superiority of serial calcitonin measurements over imaging to monitor responses to TKIs over 17 years.

## Introduction

Medullary thyroid carcinoma (MTC) accounts for approximately 2%-4% of malignant thyroid tumors.^1-3^ More than 70% of MTC cases occur sporadically while the rest are associated with MEN2A or MEN2B syndromes. Its treatment involves early total thyroidectomy and neck lymph node dissection. While preventive thyroidectomy can be curative for family members carrying *RET* mutations, sporadic cases arising from de novo mutations are often detected in advanced stages. Targeted molecular therapies discovered over the past decade have improved the prognosis of advanced cases, offering prolonged progression-free survival (PFS).^4^

Serum calcitonin concentration is a solid and sensitive biochemical marker of tumor growth used for postoperative surveillance.^5,6^ Furthermore, tumor stage and serum calcitonin are the independent prognostic parameters in MTC.^7^ Given that the natural trajectory of serum calcitonin follows a logarithmic curve in patients with MTC over time, the time required for doubling of serum calcitonin concentration (calcitonin doubling time [Ct-DT]) could reflect the progression of MTC. Indeed, the prognostic value of Ct-DT is superior to clinical staging.^8^ Since doubling time is inversely related to tumor growth, several authors have suggested using 1/Ct-DT (the slope of log-transformed calcitonin changes), the calcitonin doubling rate (Ct-DR) for disease follow-up.^8,9^ Ct-DR indicates the number of doublings (tumor growth) or halvings (shrinkage, if it is a negative value) that occur per unit of time.

In this article, we present the case of MEN2B-associated MTC with a uniquely long disease course. During the 17-year follow-up, several new tyrosine kinase inhibitors (TKIs) were used to treat our patient. Regular calcitonin surveillance over the disease course allowed us to investigate the effects of surgical interventions and TKIs on calcitonin trajectories and Ct-DR. This study was approved by the local ethics committee of Semmelweis University (SE RKEB 139/2023).

## Case Presentation

Our patient presented with a right cervical mass and characteristic features of MEN2B, including marfanoid habitus, enlarged lips, and mucosal neuromas on the tongue, at age 10 years in 2006. Fine-needle biopsy confirmed MTC; serum calcitonin was 1,597 pg/mL (reference range, 0-19). Family history and genetic analysis of the index patient and her parents confirmed de novo germline *RET* proto-oncogene mutation (Met918Thr).^10,11^

Serum calcitonin levels were monitored regularly (≥4 measurements/year; n = 114). Given that the natural increase of calcitonin levels in MTC follows a logarithmic curve over time, we modeled log-transformed calcitonin levels with the general linear model with time as the only covariate for each treatment period.^9^ The slope in this model corresponds to Ct-DR. For visual representation of the data, we estimated log-transformed calcitonin values with respective 95% confidence bands for each treatment period. For TKI inhibitors, we excluded the first 60 days of the treatment, as visual inspection of the data suggested decreasing calcitonin trajectories during this time that cannot be estimated with the above method. Furthermore, we excluded another 60 days to the end of the last TKI intake to allow the TKI effect to wane. We provide estimated Ct-DR values with 95% CIs for the following four intervals without drug treatment (A—operation 1-2, B—operation 2-3, C—operation 3-4, and D—operation 4-5) and 3 with TKI treatments (Van—vandetanib, Len—lenvatinib, and Sel—selpercatinib).

Routine radiological follow-up was performed during the whole study period with contrast enhanced magnetic resonance imaging (2011-2016: Philips Achieva 3 T, 2016-2023, Philips Ingenia 3 T). Using archived images, we systemically reanalyzed treatment response using the RECIST 1.1 criteria for the three TKI treatments lasting longer than 4 months (Van, Len, Sel). Furthermore, we provide visual representation of quantitative response data as the sum of largest diameters over the three investigated periods.

**FIG 1.**
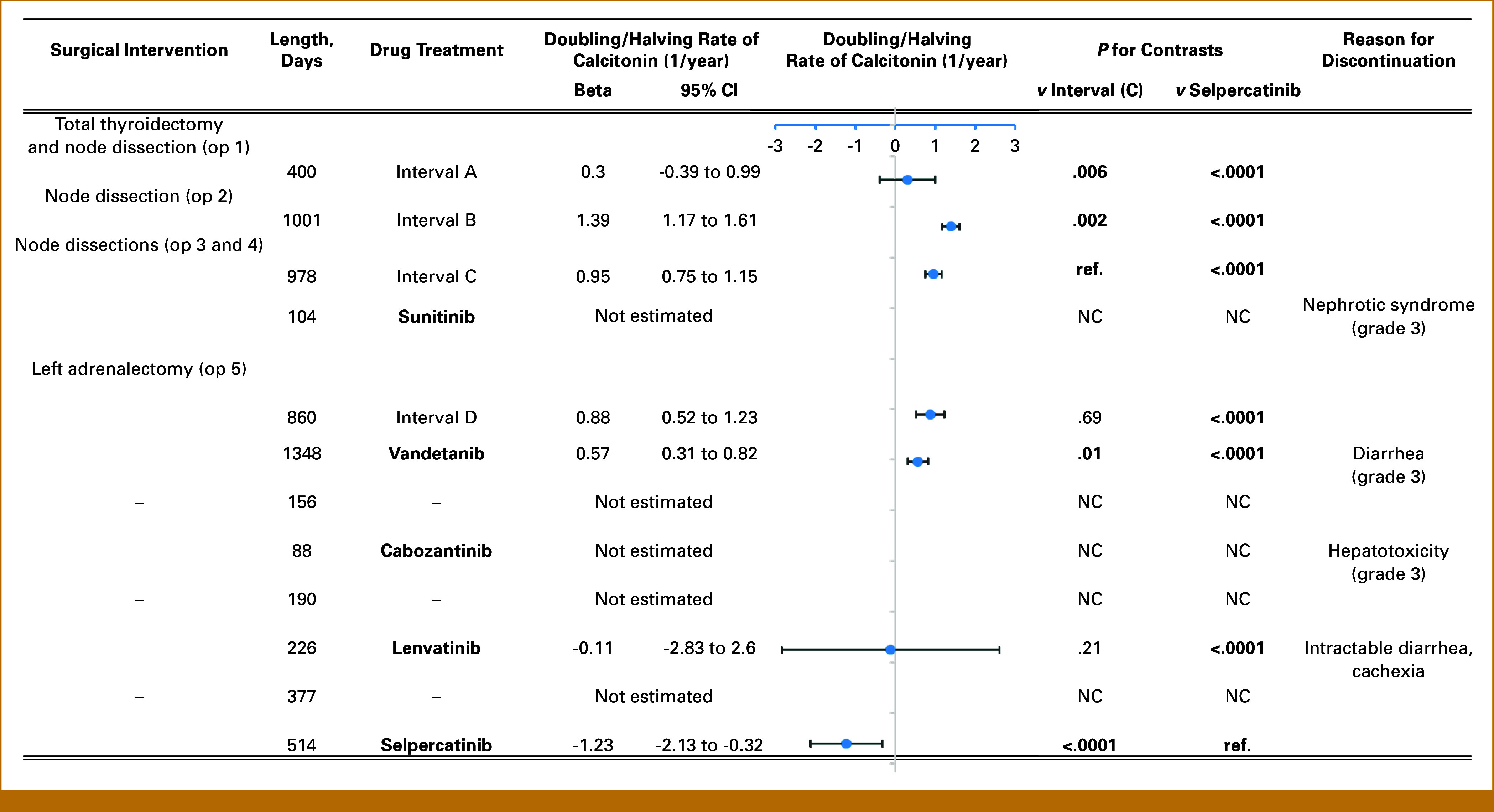
Chronology of interventions and their effect on doubling/halving rate of calcitonin. Doubling/halving rates are estimated using general linear model with log-transformed calcitonin as outcome and time as predictor. Error bars show 95% CIs. Interval A, interval B, interval C, and interval D—untreated postoperative intervals. NC, not calculated; op, operation; ref., reference.

**FIG 2.**
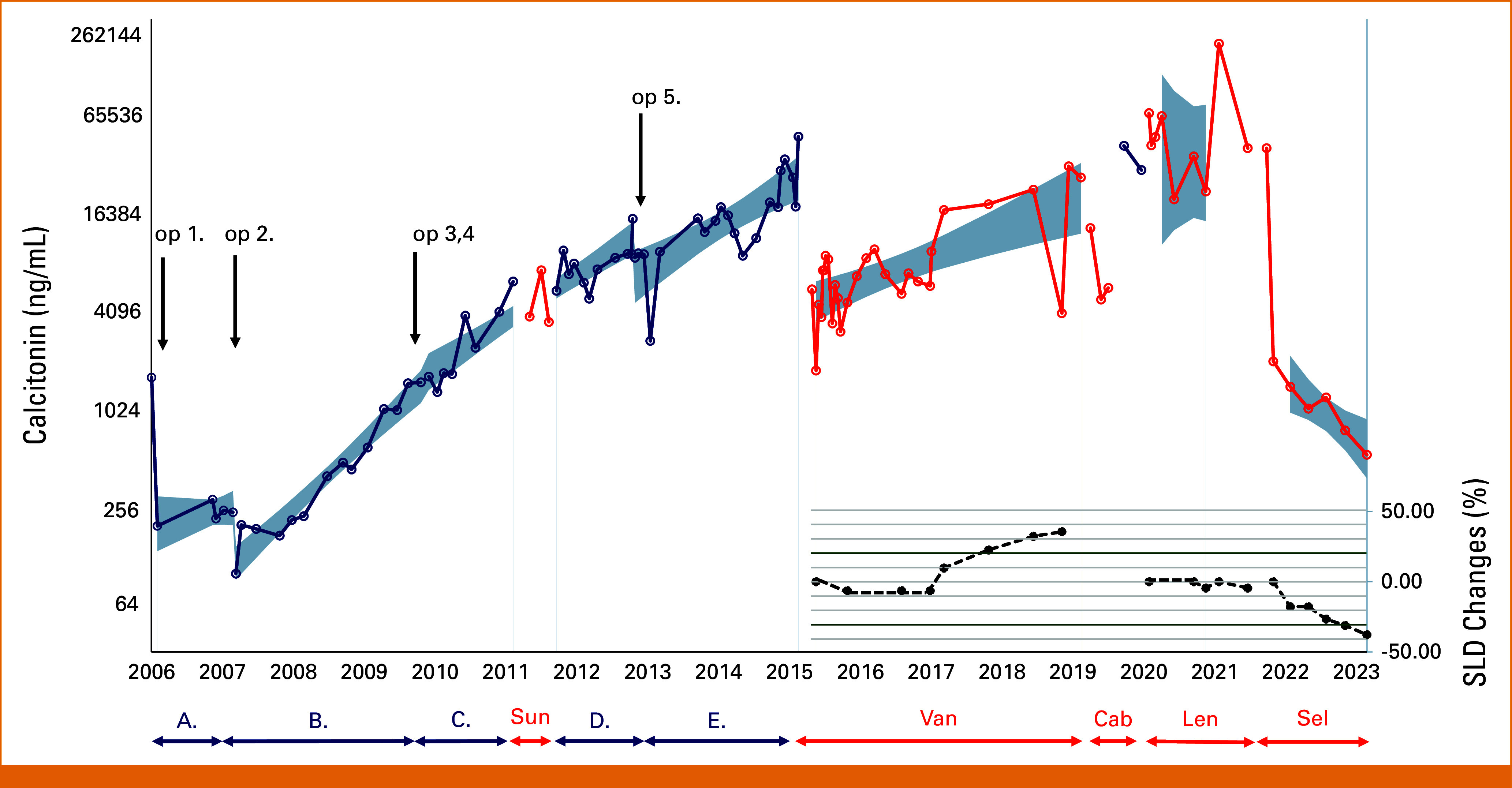
Serial serum calcitonin concentrations over 17 years of follow-up and relative changes in the SLD during Van, Len, and Sel treatments. Actual calcitonin values are plotted on a log-scale. Gray areas show 95% confidence bands of the estimated calcitonin values for a given treatment period (details in the text). Changes in SLDs are shown in the right lower insert. SLDs were expressed as percentage and set to zero at the start of each drug treatment periods with Van, Len, and Sel. The two horizontal lines at –30% and +20% indicate cutoffs of partial remission and progressive disease, as per RECIST 1.1. A, B, C, D, and E (blue line) are postoperative intervals without drug treatment. Arrows point to dates of surgical interventions (op1-op5). Cab, cabozantinib; Len, lenvatinib; op, operation; Sel, selpercatinib; SLD, sum of largest diameter; Sun, sunitinib; Van, vandetanib.

Total thyroidectomy and neck lymph node dissection were performed in April 2006. Histopathological analysis confirmed the MTC involving central and jugular regional lymph nodes (pT2aN1bMx, stage IVa). Calcitonin levels markedly decreased postoperatively from 1,597 to 193 pg/mL. In the following year, calcitonin levels showed a shallow increase (interval A) in agreement with imaging that indicated lymphoglandular involvement in the neck. After the second operation, an approximate halving of calcitonin levels was observed. However, the postoperative increase (interval B) was faster than before, reflected by the higher Ct-DR (Figs 1 and 2).

While the third and fourth operations (performed in short succession) seemed to have no significant effect on calcitonin levels immediately after the operations, the increase in calcitonin levels (Ct-DR) became shallower (interval C). In March 2011, liver metastases were found, and a short period of sunitinib treatment commenced. Sunitinib had no major effect on calcitonin levels (Figs 1 and 2).

**FIG 3.**
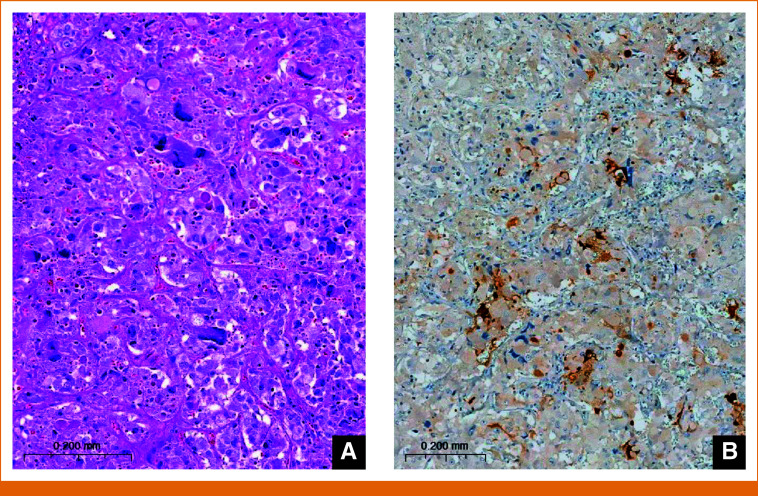
Histopathological examination of the adrenalectomy sample. (A) Diffuse growth of tumor cells with large confluent nests creating a zellballen pattern. Nuclear atypia and cellular pleomorphism are conspicuous, but there is no mitosis. (B) Focal but strong calcitonin positivity is obvious (immunostaining with calcitonin).

The last surgical intervention (January 2013) that produced a significant drop in calcitonin levels included adrenalectomy for pheochromocytoma. Although it had no significant effect on the slope of the calcitonin curve, the immediate drop probably reflects the finding that the pheochromocytoma was calcitonin-positive by histopathology (Fig 3). At the same time, the patient underwent a liver biopsy that revealed hepatic metastasis of MTC.

In the subsequent years (June 2015-December 2021), three different multikinase inhibitors (vandetanib, cabozantinib, and lenvatinib) were used to stabilize the disease course. In general, these medications necessitated frequent dose reductions and temporary treatment stops because of side effects, finally leading to their discontinuation (Table 1). Both vandetanib and cabozantinib were associated with significant drops in calcitonin levels in the first 60 days of treatment. Furthermore, vandetanib also significantly reduced the speed of calcitonin increase compared with the last untreated period (Fig 2, interval D), resulting in a more than 2-year long stable disease period as per RECIST 1.1.

**TABLE 1.** Treatment-Related AEs During Different Drug Treatments

Dose	AE	AE Grades^a^			Action Taken
		1	2	3	
Sunitinib: May 4, 2011-August 1, 2011					
37.5 mg, once daily	Rash	X			Local treatment
37.5 mg, once daily	Nausea	X			Symptomatic treatment
37.5 mg, once daily	Headache	X			Symptomatic treatment
37.5 mg, once daily	Nephrotic syndrome			X	Discontinuation of medication
Vandetanib: June 8, 2015-February 16, 2019					
100 mg, three times a day	ECG abnormal T wave	X			Dose reduction
100 mg, three times a day	ECG prolonged QT corrected interval	X			Dose reduction
100 mg, once daily	Rash	X			Interruption of medication
100 mg, three times a day	Diarrhea	X			Interruption of medication
100 mg, once daily	Rash	X			Symptomatic treatment
100 mg, twice daily	Diarrhea	X			Symptomatic treatment
100 mg, twice daily	Pericardial effusion	X			Dose reduction
100 mg, once daily	Vomiting	X			Symptomatic treatment
100 mg, once daily	Diarrhea	X			Symptomatic treatment
Cabozantinib: July 23, 2019-October 20, 2019					
140 mg, once daily	Maculo-papular rash	X			Local treatment
140 mg, once daily	Palmar-plantar erythrodysesthesia syndrome		X		Local treatment
140 mg, once daily	Epistaxis	X			Treatment not needed
140 mg, once daily	Loss of normal hair pigmentation	X			Treatment not needed
140 mg, once daily	Headache	X			Symptomatic treatment
140 mg, once daily	Increased lactate dehydrogenase level	X			Observation
140 mg, once daily	Increased AST level			X	Discontinuation of medication
140 mg, once daily	Increased ALT level			X	Discontinuation of medication
140 mg, once daily	Vomiting	X			Symptomatic treatment
Lenvatinib: April 28, 2020-November 17, 2021					
24 mg, once daily	Vomiting	X			Dose reduction
20 mg, once daily	Amenorrhea		X		Observation
20 mg, once daily	Headache	X			Symptomatic treatment
Selpercatinib: December 24, 2021-ongoing					
160 mg, twice daily	None				

Abbreviation: AE, adverse event.

^a^ Grade 4 AE did not occur.

Surprisingly, the initiation of lenvatinib had no effect on calcitonin levels in the first 60 days. However, the negative point estimate for Ct-DR suggested that lenvatinib resulted in decreasing calcitonin over the whole treatment period. This also corresponds well with the categorical radiological assessment of stable disease over the lenvatinib treatment period (Fig 2).

Despite all our efforts, the patient's somatic state continuously deteriorated. Finally, she became cachectic with secondary amenorrhea. In 2019, her body mass index was 16.9 kg/m^2^, and she complained of weakness and severe watery diarrhea (30/day). Imaging indicated further progression, with confluent liver metastases and new lytic bone lesions.

Considering the patient's deteriorating general health and that a selective RET-kinase inhibitor (selpercatinib) became available, we decided to replace lenvatinib with selpercatinib treatment in December 2021. We have used selpercatinib at its recommended starting dose without notable side effects. Selpercatinib led to a marked (>90%) decrease in calcitonin levels in the first 60 days of treatment, followed by a significantly decreasing trajectory with a slope that was lower than all previous treatment modalities. In contrast to this rapid and marked reduction of calcitonin levels, partial remission per RECIST could be diagnosed only after 12 months of treatment (Figs 1 and 2). The patient gained 10 kg in weight and became completely asymptomatic during the 22-month selpercatinib treatment.

## Discussion

Over the past one and a half decades, several TKIs have been introduced to treat advanced-stage MTCs. Multikinase inhibitors (sorafenib, sunitinib, vandetanib, and cabozantinib) have achieved moderate efficacy in MTC treatment, as reflected by increased PFS.^12-17^ However, evaluating radiological response using RECIST 1.1 criteria might be extremely difficult in advanced MTC since radiological improvement may become apparent only after several years.^17-20^

Our patient's long disease course represents typical difficulties in the real-life management of MTC in MEN2 patients. As with most patients with de novo mutations, the diagnosis was established in an advanced stage with extensive nodal involvement. The patient underwent multiple cervical lymph node dissections and the removal of a calcitonin-producing pheochromocytoma. All these operations seemed to have a significant effect on the course of the disease, reflected by either immediate drops in calcitonin levels or by decreases in the slope of postoperative calcitonin increase (Ct-DR) or a combination of the above.

The unambiguous decrease in serum calcitonin after pheochromocytoma removal and the presence of calcitonin within tumor cells were unexpected findings that suggest local calcitonin production. Calcitonin-producing pheochromocytomas are rarely reported in the literature.^21^

Altogether, four multikinase TKIs have been used to treat our patient over the past decade. By and large, drug availability was the primary determinant of the choice of subsequent drug treatment. Notably, sunitinib and cabozantinib were associated with marked biochemical response in the first 2 months after initiation, while vandetanib and lenvatinib decreased the slope of subsequent increase compared with untreated postoperative periods.

The best biochemical response was observed with the selective RET-kinase inhibitor selpercatinib, which produced the largest drop in calcitonin levels and was associated with a statistically significant decreasing trajectory of calcitonin. In the absence of notable side effects, selpercatinib also improved our patient's quality of life within a few months of treatment.

While the biochemical response to different treatment modalities was almost immediate and reflected the overall status of the patient, the radiological treatment outcomes based on either RECIST 1.1 or the SLDs happened much slower and they were overall less sensitive to the treatment effects. However, the huge effect of selpercatinib on calcitonin levels was also reflected in partial remission and as an almost 50% tumor shrinkage in terms of radiological response.

In conclusion, our patient has a 17-year survival of stage IV MTC and a 12-year survival of advanced, distant metastatic disease. Such an extended survival is rare in the literature in patients with MTC harboring the M918T mutation.^22-26^ The long disease course and regular calcitonin monitoring during the entire disease course made it possible to compare the effects of different treatment modalities, including surgical procedures and nonselective and selective TKIs. This case presents how the effectiveness of different treatment modalities can be estimated by the use of log-transformed calcitonin levels and Ct-DR and the important individual differences of the different TKIs both in terms of treatment response and side effects. Our experiences reported above support the view that serial calcitinin measurements may be more sensitive than radiological assessment per RECIST 1.1. for the follow-up of advanced MTC. Our case clearly reflects the importance of individualized therapy and illustrates the possibility of a long survival with advanced MTC. With the use of selpercatinib, our patient is approaching a 2-year PFS with excellent clinical conditions.

## Data Availability

The data of this study may be available on reasonable request to the corresponding author.
